# Evaluation and associated risk factors for neutropenia with venetoclax and obinutuzumab in the treatment of chronic lymphocytic leukemia

**DOI:** 10.1002/cnr2.1505

**Published:** 2021-07-12

**Authors:** Courtney Samuels, Diana Abbott, Sierra Niemiec, Jennifer Tobin, Angela Falco, Keri Halsema, Manali Kamdar

**Affiliations:** ^1^ Blood Disorders Center University of Colorado Aurora Colorado USA; ^2^ Center for Innovative Design and Analysis, Department of Biostatistics and Informatics University of Colorado‐Anschutz Aurora Colorado USA

**Keywords:** chronic lymphocytic leukemia, myelosuppression, pharmacotherapy, toxicity, venetoclax

## Abstract

**Background:**

The time‐limited combination of venetoclax and obinutuzumab (VenG) was established by the German CLL Study Group in the CLL14 trial for the upfront management of newly diagnosed chronic lymphocytic leukemia (CLL), showing a superior progression free survival benefit. The incidence of grade 3–4 neutropenia was reported in the range of 52.8%–57.7%. However, patients who develop neutropenia with this combination have yet to be formally characterized in the literature as it has impact on the clinical practice setting.

**Aim:**

To determine the incidence of grade 3 and 4 neutropenia and identify risk factors for neutropenia among CLL patients treated with the VenG regimen.

**Methods:**

We conducted a retrospective, single‐center study of all adult patients with a diagnosis of CLL treated with VenG at the University of Colorado Hospital. Demographic information, laboratory data, clinical data, and medication prescriptions were collected from the patients' electronic medical record.

**Results:**

A total of 14 patients (73%) developed neutropenia during the course of therapy. The mean time to neutropenia from the start of treatment was 42 days (range 1–131). Our cohort harbored more high risk disease features and more comorbidities (CIRS score of 12). Four patients (28.6%) in the neutropenic group developed infectious complications during therapy and 6 (31%) patients were unable to be dose escalated to the final FDA approved dose of 400 mg.

**Conclusion:**

Our study cohort had higher incidence of grade 3 and 4 neutropenia occurring in 73% of patients. This could be attributed to a higher rate of comorbidities, high risk features, concomitant interacting medications, and prior chemotherapy. Further studies are warranted to determine if growth factor support is efficacious to achieve dose escalation with this therapy.

## 1 INTRODUCTION

Treatment of Chronic Lymphocytic Leukemia (CLL) has radically transformed over the past decade with the advent of targeted therapies. The addition of these molecularly targeted therapeutics, as compared to chemotherapy agents, have improved survival and tolerability.^1^, ^2^, ^3^, ^4^ Venetoclax is an oral selective B‐cell lymphoma 2 (BCL2) inhibitor, an important antiapoptotic protein expressed on B‐cells. Venetoclax promotes cell death by disrupting antiapoptotic cell signaling.^5^, ^6^ Obinutuzumab, an anti‐CD20 antibody, in combination with venetoclax (VenG) was established by the German CLL Study Group in the CLL14 trial, showing a superior progression free survival benefit.^1^ This combination has been an attractive option for patients as it allows for a fixed duration of treatment when compared to the continuous administration necessary for other targeted oral agents, including the Bruton's tyrosine kinase inhibitors and the phosphatidylinositol‐3 kinase inhibitors.^1^, ^4^

While venetoclax is a more selective BCL2 inhibitor, lacking the dose limiting thrombocytopenia of its predecessor navitoclax, neutropenia remains a concern.^6^, ^7^, ^8^ Neutropenia has been reported in the phase 3 studies. The incidence of grade 3–4 neutropenia reported in CLL‐14 was reported to be 52.8%, and 58% at long term follow up.^1^, ^9^, ^10^ By contrast, the MURANO trial examined the use of an anti‐CD20 agent with venetoclax in the relapsed refractory setting and found the incidence of grade 3–4 neutropenia to be 57.7%.^4^ Data with respect to real world incidence of neutropenia is sparse. Davids et al report their experience with venetoclax monotherapy in the relapsed/refractory setting and characterize additional risk factors in this population. This analysis found patients, classified as high risk (Binet stage C and a neutrophil count <5.64 × 10^9^/L or Binet stage A/B and neutrophil count <2.46 × 10^9^/L), to have a reported rate of grade 3 and 4 neutropenia of 83%.^11^ However, patients who develop neutropenia with this combination have yet to be characterized in the literature outside the context of a clinical trial. Understanding the clinical characteristics of neutropenia in the clinic setting for patients undergoing therapy with VenG would assist hematologists in their day‐to‐day practice.

The objective of this study was to determine the incidence of grade 3 and 4 neutropenia and identify risk factors for neutropenia among CLL patients treated with the VenG regimen.

## 2 PATIENTS AND METHODS

### 2.1 Study design

We conducted a retrospective, single‐center study of all adult patients with a diagnosis of CLL treated with VenG. This included all patients aged 18 years or older at the University of Colorado Hospital from July 2019 to the data cutoff of March 2021. Demographic information, laboratory data, clinical data, and medication prescriptions were collected from the patients' electronic medical record. This study was approved by the University of Colorado Hospital Institutional Review Board (IRB#20‐2774).

### 2.2 Patients

Patients were included if they had a diagnosis of CLL and were treated with the regimen of VenG. Patients could have received treatment in the frontline or relapsed/refractory setting but must have received venetoclax in combination with obinutuzumab. Patients with incomplete medical records were excluded.

### 2.3 Data collection

Demographic and clinical data were collected from the patients' electronic medical record including age, gender, race, prior radiation history, and number of prior therapies. Prognostic factors collected included Rai stage, immunoglobulin heavy chain rearrangement status, lymph node size, next generation sequencing, and fluorescence in situ hybridization testing. Pertinent labs were recorded immediately prior to VenG initiation (Table 1) and followed until end of therapy. Neutropenia in this study was defined as an absolute neutrophil count less than 1000/mm^3^ as defined by the Common Terminology Criteria for Adverse Events.^9^ Prior medication data including concomitant cytochrome P450 (CYP) 3A4 inhibitors and inducers, p‐glycoprotein (P‐gP) inhibitors, as well as granulocyte colony stimulating factors were captured. Descriptive statistics were used for all outcomes.

**TABLE 1:** Baseline Labs

Baseline lab	(*n* = 19) median (range)
WBC	32.8 (1.5–302.4)
Hgb	11.61 (6.1–15)
Platelets	153.05 (38–310)
Scr	1.01 (0.65–1.88)
AST	17 (8–33)
ALT	29 (15–76)
Total bilirubin	0.7 (0.4–1.1)
LDH	435.74 (149–942)

Abbreviations: AST, aspartate transaminase; ALT, alanine aminotransferase; Hgb, hemoglobin; LDH, lactate dehydrogenase; WBC, white blood cell count.

## 3 RESULTS

### 3.1 Patient characteristics

We identified 19 patients eligible for review who were predominantly Caucasian (94.7%) and male (57.9%). The patient demographics at initiation of VenG can be found in Table 2. The median age at initiation was 63 years with 42.1% having Rai Stage III or IV disease and a median Cumulative Illness Rating Scale (CIRS) score of 12 (range of 5–22). Five patients (26.3%) harbored a 17p deletion and 5 patients (26.3%) had deletion 11q. Immunoglobulin heavy chain rearrangement unmutated were seen in a majority of patients (89.5%) and five patients (26.3%) carried a Tp53 mutation. VenG was predominantly the first line therapy in the overall study population, while 7 patients (36%) received prior chemotherapy (range 0–2). There were no patients with prior radiation therapy. Labs immediately prior to initiation of therapy, including organ function are described in Table 1.

**TABLE 2:** Patient Demographics

Patient characteristic	Overall (*n* = 19), *n*(%)
Age at onset of therapy, y	
Median	63
Range	(50–81)
Gender	
Male	11 (57.9%)
Race/Ethnicity	
Caucasian	18 (94.7%)
Vietnamese	1 (5.3%)
Rai stage	
Stage 0	1 (5.3%)
Stage 1	8 (42.1%)
Stage 2	2 (10.5%)
Stage 3	3 (15.8%)
Stage 4	5 (26.3%)
Prior lines of therapy	
0	12 (63.2%)
1	6 (31.6%)
2	1 (5.3%)
Lymph node size (cm)	
Median	2.0
Range	0.5–5.1
CIRS score	
Median	12
Range	(5–22)
Molecular features	
17p deletion	5 (26.3%)
11q deletion	5(26.3%)
Deletion 13	4 (21.1%)
6q deletion	1 (5.3%)
Trisomy 12	3 (15.8%)
IGHV	
Unmutated	17 (89.5%)
Mutated	2 (10.5%)
TP53	
Mutated	5 (26.3%)
Unmutated	14 (73.7%)

Abbreviations: CIRS, Cumulative Illness Rating Scale; IGHV, Immunoglobulin Heavy Chain Variable Region Gene.

### 3.2 Neutropenia

A total of 14 patients (73%) developed neutropenia during the course of therapy. The median time to neutropenia from the start of treatment was 42 days (range 1–131). Seven patients received prior therapy and six of those patients (86%) developed neutropenia. This is in contrast to those who received VenG as upfront therapy in which eight patients (66%) experienced neutropenia. A summary of these outcomes are listed in Table 3.

**TABLE 3:** Outcomes

Outcomes	Patients N = 19
Overall Grade 3–4 Neutropenia N(%)	14 (73.7)
Grade 3–4 Neutropenia in Relapsed Refractory (*n* = 7)	6 (85.7)
Grade 3–4 Neutropenia in Frontline Therapy (*n* = 12)	8 (66.7)
Time to neutropenia, median, days	42 (1–131)
Dose reduction or interruption N(%)	6 (31)
Appropriateness of dose reduction N(%)	3 (15.8)
Infectious complications N(%)	5 (26.3)
Growth factor support N(%)	6 (31.6)
Venetoclax dose at neutropenia (mg)	20 (20–400)
Concurrent CYP3A4^a^ or p‐gP^b^ inhibitors N(%)	6 (31.6)

^a^ CYP, cytochrome P450,

^b^ p‐gP, p‐glycoprotein.

Four patients (28.6%) in the neutropenic group developed infectious complications during therapy, as compared to one patient in the non‐neutropenic group. This included two (10%) bacterial pneumonias, one (5%) septic arthritis, one (5%) fungal infection, and one (5%) *Pneumocystis jirovecii* pneumonia. In the overall study population eight (42%) patients experienced dose reductions or dose interruptions for neutropenia. In all, six (31%) patients were unable to be dose escalated to the final FDA approved dose of 400 mg. The reasons for halting dose escalation included concomitant drug interactions (*n* = 2; 10%), infections (*n* = 2; 10%), and neutropenia (*n* = 2; 10%). Of those who developed neutropenia, five patients (28.6%) experienced neutropenia within the first 21 days of therapy, this would coincide during the Obinutuzumab load period of therapy. Nine patients (64.3%) developed neutropenia on or after day 22, during the venetoclax escalation, and the median dose at which neutropenia developed was 20 mg. Those with neutropenia had a median white blood cell (WBC) count of 42.19 × 10^9^/L prior to therapy initiation and non‐neutropenic patients had a median WBC count of 161.16 × 10^9^/L. The median absolute neutrophil count at therapy initiation was 2.6 × 10^9^/L. However, lymphocytosis was seen in 12 patients (63.2%) prior to initiation suggesting a majority of patients may be functionally neutropenic.

### 3.3 Concomitant medications

In all, six (31%) patients received growth factor support. Four (21%) patients received filgrastim, one (5%) received pegfilgrastim, and one (5%) received both filgrastim and pegfilgrastim support during their treatment. Use of a moderate CYP3A4 inhibitors and P‐gP inhibitors occurred in six (31%) patients with three receiving venetoclax dose reductions as recommended per the prescribing information. The moderate CYP3A4 inhibitors included fluconazole (*n* = 4; 15%), isavuconazonium (*n* = 1; 5%), and diltiazem (*n* = 1; 5%). One (5%) patient received a P‐gP inhibitor, amiodarone, with concomitant fluconazole.

## 4 DISCUSSION

The prior published studies describing phase 3 data utilizing an anti‐CD20 agent with venetoclax have described associated adverse effects including neutropenia. However, this data was gathered in the context of clinical trials which may not be representative of the general CLL population. To our knowledge this is the only reported series to describe neutropenia associated with this regimen as it relates to clinical practice. The incidence described by prior studies state grade 3 and 4 neutropenia to be 52.8%–58%.^1^, ^4^, ^10^ Fischer and colleagues evaluated VenG in the frontline setting, particularly in those with coexisting conditions (CIRS score ≥6), and found 52.8% of patients experienced grade 3 and 4 neutropenia.^1^ The published data at extended follow up (CLL‐14) revealed that 58% of patients experienced grade 3–4 neutropenia in the front‐line setting.^10^ By contrast, Seymour et al examined venetoclax and an alternative anti‐CD20 agent, rituximab, in the relapsed refractory setting showing only 43.3% of patients with grade 3–4 neutropenia.^4^ However, the rate observed in our cohort is higher than previous reports with 66% of patients experiencing grade 3–4 neutropenia with up front therapy, 86% of patients in the relapsed refractory setting, and 73% in the overall cohort.

The variation in the reported incidence is likely multifactorial, including the differences in the study patient populations. Individual risk factors for neutropenia in the cancer population have been previously elucidated.^12^, ^13^ Some of these individual risk factors include advanced age, presence of comorbidities, prior chemotherapy or radiation, bone marrow involvement, pre‐existing neutropenia and organ dysfunction.^14^ Our patient cohort, as compared to the aforementioned studies, harbored more high‐risk features including del 17p (26.3%), unmutated IGHV (89.5%), and TP53 mutated (26.3%). This also coincided with a high number of comorbidities as measured by the CIRS score with 94.7% of patients with a CIRS score ≥ 6. Additionally, 36.8% of our cohort received at least one prior line of therapy prior to VenG treatment. Furthermore, pre‐existing neutropenia is an established risk factor and while the median WBC in our review was 38.3 × 10^9^/L (1.5–302.4 × 10^9^/L) it is plausible to assume many were functionally neutropenic which is not reflected by laboratory parameters. This was further demonstrated by the infectious complications (26.3%) seen in both neutropenic and non‐neutropenic patients as defined by laboratory parameters.

The addition of monoclonal antibodies, such as anti‐CD20 agents, have been known to be an independent factor for neutropenia both with and without chemotherapy.^14^, ^15^ This should be considered as a compounding factor for higher rates of neutropenia in this setting. Obinutuzumab also appears to be associated with higher rates of neutropenia as compared to its counterpart, rituximab.^15^ This was reflected in our cohort with a median onset to neutropenia of 42 days and at a median venetoclax dose of 20 mg. Additionally, consideration should be taken when using these antibodies in the relapsed/refractory setting as studies have shown a higher incidence of neutropenia when compared to patients in the frontline setting.^16^, ^17^ Prescribing information for venetoclax have specified dose modifications for hematologic toxicities, however the recommendations provided for Obinutuzumab are less distinct.^5^, ^18^, ^19^ Management of neutropenia in this setting can involve dose interruption, reduction and/or growth factor support.^20^ A post hoc analysis of the MURANO study found that venetoclax treatment dose reduction or interruption for adverse effects had no effect on progression free survival outcomes.^20^ In contrast, treatment discontinuation was associated with inferior PFS outcomes. This highlights the importance of prompt management of adverse effects, including neutropenia, to maximize treatment duration and avoid therapy discontinuation.^20^ Furthermore, currently no guideline exists for initiation of growth factor in this particular population but is left to the discretion of the provider.^14^ 31.6% of our cohort received growth factor support, and of those receiving growth factor (*n* = 6), all but one patient was able to complete the venetoclax dose escalation to the final 400 mg dose. The small sample size and retrospective nature of our study makes it difficult to extrapolate the benefit of this approach to a wider population, but does warrant further study.

Finally, additional consideration should be made regarding the effect of concomitant medications, particularly agents affecting CYP3A4 or P‐gP. Azole antifungals are used for prevention of fungal infections in at risk patients, but are also known inhibitors of CYP3A4. Moderate inhibitors, such as fluconazole, would require a 50% dose reduction in venetoclax per the prescribing information.^5^ Our retrospective chart review had a low‐rate azole prophylaxis (*n* = 4), and 31% (*n* = 6) of patients overall in our study received concomitant CYP3A4 or p‐gP inhibitors. Only three patients did not have dose adjustments as recommended per the prescribing information, which did not allow for meaningful comparisons. However, prescribing of such agents especially during the dose escalation of venetoclax should be taken with caution and the appropriate dose adjustment, as this could lead to unwarranted neutropenia. Additionally, concomitant medications that have myelosuppressive potential, such as trimethoprim/sulfamethoxazole, should be used with careful consideration as this may be a compounding factor in the development of neutropenia. Two patients within our cohort received concomitant trimethoprim/sulfamethoxazole during the treatment period making it difficult to extrapolate the impact this may have on count suppression. Future studies are required to answer this question and what effect concomitant medication interactions may have on outcomes and incidence of neutropenia.

Our study has several limitations. First, our cohort is from a single center retrospective review and was predominantly composed of Caucasian males. As a result, it may be difficult to generalize to a broader population with more diverse demographics. Secondly, the small sample size of our study led way for descriptive statistics but was not powered to account for confounders. It should also be noted the use of growth factor during the study period was at the discretion of the treating provider. Lastly, because a retrospective chart review requires heavy reliance on adequate documentation the use of over‐the‐counter medications may have been under reported.

## 5 CONCLUSIONS

Our retrospective study showed an increase incidence of grade 3 and 4 neutropenia associated with this regimen as compared to previous reports. Our study cohort had higher rates of comorbidities, high risk features, concomitant interacting medications, and prior chemotherapy. Providers should be mindful of concomitant interacting medications that could increase drug exposure with this regimen or cause compounding neutropenia. Further studies are warranted to determine if proactive growth factor support is efficacious to maximize therapy duration, as well as prospective studies to assess the impact of dose interruption and modification on overall survival.

## CONFLICT OF INTEREST

Manali Kamdar reports research funding from TG Therapeutics, Genetech and personal fees from AbbVie, KaryoPharm, Kite, AstraZeneca, Celgene/Bristol‐Myers Squibb, Adaptive Biotechnologies, and ADC therapeutics. The authors report no other conflicts of interest.

## AUTHORS' CONTRIBUTIONS

All authors had full access to the data in the study and take responsibility for the integrity of the data and the accuracy of the data analysis. *Conceptualization*, S.C., T.J., H.K. and K.M.; *Methodology*, S.C., A.D., T.J., F.A., H.K. and K.M.; *Investigation*, S.C., T.J., F.A., H.K. and K.M.; *Formal Analysis*, A.D., N.S. and K.M.; *Resources*, F.A. and K.M.; *Writing‐Original Draft*, S.C.; *Writing‐Review Editing*, S.C., T.J. and K.M.; *Visualization*, H.K.; *Supervision*, S.C. and K.M.; *Data Curation*, S.C., A.D., N.S. and F.A.; *Project Administration*, S.C., F.A. and H.K.; *Software*, A.D. and N.S.

## ETHICAL STATEMENTS

University of Colorado Institutional Review Board (IRB#20‐2774) waived the need for ethics approval and the need to obtain consent for the collection, analysis and publication of the retrospectively obtained and anonymized data for this non‐interventional study.

## Data Availability

The data that support the findings of this study are available on request from the corresponding author. The data are not publicly available due to privacy or ethical restrictions.
